# The Long-term Volumetric and Radiological Changes of COVID-19 on Lung Anatomy: A Quantitative Assessment

**DOI:** 10.2174/0115734056372497250716235052

**Published:** 2025-07-24

**Authors:** A. Savranlar, M. Öztürk, H. Sipahioğlu, Y. Savranlar, M. Tahta Şahingöz

**Affiliations:** 1 Department of Radiology, Kayseri City Training and Research Hospital, Kayseri, Türkiye; 2 Department of Anatomy, Kayseri City Training and Research Hospital, Kayseri, Türkiye; 3 Department of Intensive Care, Kayseri City Training and Research Hospital, Kayseri, Türkiye; 4 Department of Medical Histology and Embryology, Kayseri City Training and Research Hospital, Kayseri, Türkiye; 5 Department of First and Emergency Aid Program, Niğde Ömer Halisdemir University, Niğde, Türkiye

**Keywords:** COVID-19, Lung volume, Radiological scoring, Pulmonary fibrosis, 3D Slicer, SARS-CoV-2

## Abstract

**Objective:**

This study aimed to assess the long-term volumetric and radiological effects of COVID-19 on lung anatomy. The severity of the disease was evaluated using radiological scoring, and lung volume measurements were performed *via* 3D Slicer software.

**Methods:**

A retrospective analysis was conducted on a total of 127 patients diagnosed with COVID-19 between April 2020 and December 2023. Initial and follow-up chest CT scans were reviewed to analyze lung volumes and radiological findings. Lung lobes were segmented using 3D Slicer software to measure volumes. Severity scores were assigned based on the Chung system, and statistical methods, including logistic regression and Wilcoxon signed-rank tests, were used to analyze outcomes.

**Results:**

Follow-up CT scans showed significant improvements in lung volumes and severity scores. The left lung total volume increased significantly (p = 0.038), while right lung total volume and COVID-19-affected lung volumes demonstrated non-significant improvements. Severity scores and the number of affected lobes decreased significantly (p < 0.05). Correlation analyses revealed that age negatively influenced lung volume recovery (r = -0.177, p = 0.047). Persistent pathological findings, such as interstitial thickening and fibrotic bands, were observed.

**Conclusion:**

COVID-19 induces lasting changes in lung structure, particularly in elderly and severely affected patients. Long-term follow-up and the consideration of antifibrotic therapies are essential to manage post-COVID-19 complications effectively. A multidisciplinary approach is recommended to support patient recovery and minimize healthcare burdens.

## 1 INTRODUCTION

Coronavirus Disease 2019 (COVID-19) is a contagious respiratory illness caused by severe acute respiratory syndrome coronavirus 2 (SARS-CoV-2), which affects humans. First identified in Wuhan, China, in 2019, the disease rapidly spread worldwide. The World Health Organization (WHO) declared COVID-19 a pandemic in March 2020, designating it a Public Health Emergency of International Concern (PHEIC). As of June 20, 2024, WHO data reported 775,583,309 cases and 7 million deaths globally [1-3].

### 1.1 COVID-19 Diagnosis and the Role of Imaging Modalities

The diagnosis of COVID-19 is typically established through RT-PCR testing of oropharyngeal and nasopharyngeal swabs, with chest CT scans playing a crucial role. During the diagnostic process, various imaging features, including focal ground-glass opacities, the crazy-paving pattern, and consolidations, have been identified [4-6].

### 1.2 Effects of COVID-19 on Lung Anatomy

The lungs consist of two main structures: the right and left lungs, each composed of lobes and segments. The right lung has three lobes (upper, middle, and lower), while the left lung has two lobes (upper and lower). These lobes are interconnected *via* the bronchial branching system, enabling gas exchange through alveolar structures [7]. COVID-19 infection can cause changes in these anatomical structures, including interstitial inflammation, fibrosis, alveolar damage, and consolidation, often presenting with radiological involvement at the lobar level [4, 8].

### 1.3 Effects on the Lungs and Long COVID-19

COVID-19 primarily spreads *via* respiratory transmission, with key symptoms including fever, cough, shortness of breath, headache, and loss of taste and smell. The disease can severely impact the lung parenchyma, causing interstitial inflammation, consolidation, and damage. Post-treatment, persistent changes in the lungs and Long COVID-19 syndrome manifest with symptoms, such as fatigue, dyspnea, and cognitive impairments [9-12].

Numerous studies have reported decreased diffusion capacity and reduced respiratory muscle strength during the early recovery phase post-COVID-19 infection. While clinical tests (*e.g.*, Diffusion Capacity for Carbon Monoxide, Spirometry, and the 6-Minute Walk Test) are commonly used to evaluate pulmonary impairment in Long COVID-19 patients, imaging modalities hold significant importance. Chest CT scans are considered the most effective radiological test for diagnosing and monitoring COVID-19 patients. Studies have demonstrated a wide range of acute radiological findings, from mild to severe, during the disease's acute phase. Additionally, more than half of COVID-19 patients show fibrosis-like changes (*e.g.*, ground-glass opacities, consolidations, pulmonary interstitial thickening, and the crazy-paving pattern) persisting beyond the recovery phase (30 days post-acute infection) [8, 13, 14]. Pulmonary fibrosis is one of the most significant long-term consequences of COVID-19. Studies have shown that approximately 30% of patients who experience severe COVID-19 develop persistent fibrotic changes. This condition negatively impacts patients' quality of life and adversely affects respiratory function [15].

### 1.4 Study Objective and Importance

The primary objective of this study is to assess the severity of COVID-19 in patients treated as outpatients or inpatients through radiological scoring methods and evaluate lung volume measurements using the 3D Slicer program [16-18]. These measurements are analyzed in relation to pneumonia findings and lung volume data obtained from follow-up chest CT scans during the recovery phase. This study aims to contribute to the understanding of post-treatment volumetric changes and pneumonia findings in the lungs of COVID-19 patients.

## 2 MATERIALS AND METHODS

### 2.1 Ethical Approval

Ethical approval for this study was obtained from the Clinical Research Ethics Committee of Kayseri City Hospital on October 31, 2023 (decision no. 936). The study adhered to the principles of the Helsinki Declaration, Sager guidelines were followed.

### 2.2 Study Design and Population

This study was designed as an observational retrospective analysis to quantitatively examine lung volume changes in patients recovering from COVID-19. The study population comprised patients who presented with COVID-19 symptoms at Kayseri City Education and Research Hospital between April 28, 2020, and December 26, 2023, tested positive *via* RT-PCR, and exhibited typical COVID-19 chest CT findings. Follow-up chest CT scans were obtained as part of routine clinical evaluations. Due to the retrospective nature of the study, the sample size was determined by the number of eligible patients meeting the inclusion criteria within the defined period. Power analysis was not performed due to this retrospective design.

### 2.3 Inclusion Criteria

- Symptomatic patients diagnosed with COVID-19 *via* RT-PCR and exhibiting CT findings suggestive of viral pneumonia.
- Patients who attended routine follow-ups and were deemed suitable for tomography by their clinical physician, due to the retrospective nature of the study.

### 2.4 Exclusion Criteria

- Patients with entirely normal CT findings.
- Patients with known chronic obstructive pulmonary disease (COPD), prior lung surgeries, or lung masses, as well as those exhibiting inflammatory and/or fibrotic lung changes.
- Patients unable to comply with breath-holding instructions during CT imaging, resulting in motion artifacts.

### 2.5 Data Collection Method

In this retrospective study, patient data were retrieved from medical records. Radiological scores were assessed using the Chung system [19], and volume measurements were conducted using the 3D Slicer software [16-18, 20]. The study included a total of 127 patients, and demographic information (gender, age), treatment modality (outpatient/inpatient), hospital stay duration, and mortality status were recorded.

### 2.6 Imaging Methodology

Initial and follow-up tomography images were obtained within a defined period (median 54 days, IQR: 37–98) after the COVID-19 diagnosis. The retrospective nature of the study and variations in follow-up schedules contributed to the wide IQR of 37-98 days. These images were evaluated for lung volume and functional changes and scored by two radiologists.

### 2.7 Segmentation and Observer Reliability

Lung segmentation was performed using 3D Slicer software by two independent radiologists. To ensure consistency and accuracy, an inter-observer reliability analysis was conducted, yielding a Cohen’s kappa coefficient of 0.89, indicating strong agreement. Any discrepancies between segmentations were resolved through consensus discussions. Before the study, both radiologists underwent a standardized training program on the use of 3D Slicer software to minimize variability. This training included segmentation protocols, region-of-interest selection, and handling of borderline cases.

### 2.8 COVID-19 Lung Segmentation Procedure

For lung segmentation and volumetric analysis, the LungCTAnalyzer extension of 3D Slicer software was utilized. The segmentation process consisted of five main steps [16-18]:

#### 2.8.1 Data Import and Preprocessing

Chest CT scans in DICOM format were imported into 3D Slicer software. During this process, necessary preprocessing steps, such as noise reduction and intensity normalization, were applied to enhance image quality and ensure consistency across scans.

#### 2.8.2 Automated Lung Segmentation

The LungCTAnalyzer module was employed to segment lung structures automatically. This tool utilizes threshold-based segmentation algorithms to distinguish between healthy lung parenchyma, infiltrative areas, and fibrotic changes. The automatic segmentation process provided an initial lung volume assessment, which was further refined to enhance accuracy.

#### 2.8.3 Manual Refinement

Following automated segmentation, two independent radiologists reviewed the segmentations to ensure accuracy. If any discrepancies were identified, manual corrections were performed using the Segment Editor module of 3D Slicer. This step was particularly important for refining boundary accuracy and eliminating artifacts that could affect volumetric measurements.

#### 2.8.4 Quantification and Analysis

After segmentation, volumetric measurements were extracted to determine changes in total lung volume and COVID-19-affected regions. The segmented areas were analyzed using standardized scoring systems, allowing for an objective classification of lung involvement severity.

#### 2.8.5 Visualization and Reporting

To facilitate qualitative assessment, 3D renderings of the segmented lungs were generated. These visual outputs provided a detailed representation of lung involvement, aiding both in clinical interpretation and statistical analysis. The final segmentation results were then exported for further evaluation and data processing.

This workflow ensured standardized and reproducible lung segmentation, contributing to the quantitative assessment of COVID-19-related pulmonary changes [16-18, 20].

### 2.9 Determination of Lung Volume and Functional Changes

**Fig. (1):**
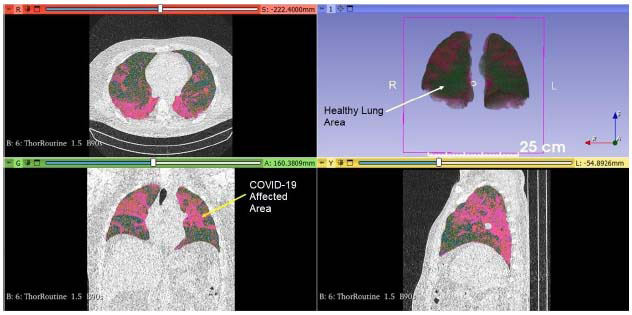
Visualization of lung volumes measured using 3D Slicer software in a patient with lung involvement due to COVID-19. Pink areas indicate regions affected by COVID-19.

Using 3D Slicer software, each lobe of the lungs (upper, middle, and lower lobes for the right lung; upper and lower lobes for the left lung) was analyzed individually. Additionally, a segmental evaluation of the affected areas was conducted, enabling a quantitative assessment of radiological changes in each segment and lobe. The total lung volume and the volume of COVID-19-affected regions were measured. Data from the initial and follow-up CT scans were compared to determine volumetric changes in the lungs (Fig. **1**).

The following parameters were evaluated:

- Right Lung Total Volume
- Left Lung Total Volume
- Total Lung Volume Affected by COVID-19

### 2.10 Radiologist Scoring

The five lung lobes were assessed for the degree of airspace opacity (GGO/consolidation) due to disease using the scoring model developed by Chung *et al.* [19] and modified by Dhoot *et al.* [20]:

- **Score 1:** <5% involvement.
- **Score 2:** 5–25% involvement.
- **Score 3:** 26–49% involvement.
- **Score 4:** 50–75% involvement.
- **Score 5:** >75% involvement.

The total CT involvement score (BT-IS) ranged from 0 (no involvement) to 25 (maximum involvement across all five lobes exceeding 75%). Based on this scale:

- Scores 0–9 indicated mild disease,
- Scores 10–17 indicated moderate disease,
- Scores 18–25 indicated severe disease [20].

To prevent bias, radiologists were blinded to patient identities and clinical outcomes during the scoring process. All CT scans were anonymized before evaluation, and a blinded review protocol was implemented. Additionally, inter-rater reliability was assessed to ensure consistency across different radiologists, demonstrating high agreement in scoring classifications.

### 2.11 Statistical Analysis

Data analysis was performed using the SPSS statistical software. The normality of data distribution was assessed using numerical methods, including the Shapiro-Wilk test and skewness-kurtosis values, as well as graphical methods such as Q-Q plots and histograms. Since the data followed a normal distribution, a Paired-Sample T-Test was conducted to evaluate the significance of differences between the arithmetic means of two related groups. Pearson Correlation Analysis was performed to examine the relationships between variables such as age, length of hospital stay, time interval between imaging, total score differences, and volume differences between the right and left lungs. Additionally, Binary Logistic Regression analysis was conducted to identify factors influencing mortality. Statistical significance was determined as a p-value <0.05.

## 3 RESULTS

### 3.1 Demographic Findings of Patients

A total of 127 patients were included in the study, of whom 62% were male and 38% were female. Among the patients, 88% received inpatient treatment, while 12% were treated as outpatients. The mean age was 55.6 ± 15.89 years, with a median hospital stay of 11 days (IQR: 6–19 days). The median time between initial and follow-up CT scans was 54 days (IQR: 37–98 days). Mortality occurred in 11% of the patients, distributed as follows:

- **Severity Scale 1:** 78 patients, 14 deaths (17.95% mortality rate),
- **Severity Scale 2:** 34 patients, 11 deaths (32.35% mortality rate),
- **Severity Scale 3:** 15 patients, 3 deaths (20.00% mortality rate).

### 3.2 Lung CT Findings

Volumetric analyses using the 3D Slicer software determined the total volumes of the right lung, left lung, and overall lung.

- The left lung total volume showed a statistically significant increase in follow-up measurements compared to initial measurements (p = 0.038).
- Although not statistically significant, increases were observed in the right lung total volume, left lung COVID-19-affected volume, and total lung volume. Decreases were noted in the right lung COVID-19-affected volume and the total volume of lung regions affected by COVID-19 (Table **1**).

### 3.3 Findings: Radiologist Scoring Results

The scoring results performed by radiologists showed significant improvements in the right and left lungs between initial and follow-up CT scans. Additionally, the severity scale demonstrated noticeable progress between these periods (Table **2**).

### 3.4 Scoring Results and Number of Affected Lobes

The scoring outcomes of the right lung, left lung, and total lung, along with the number of affected lobes, were assessed. Follow-up measurements showed a significant decrease in scores compared to initial measurements for the right lung, left lung, and total lung (p < 0.05).

**Fig. (2):**
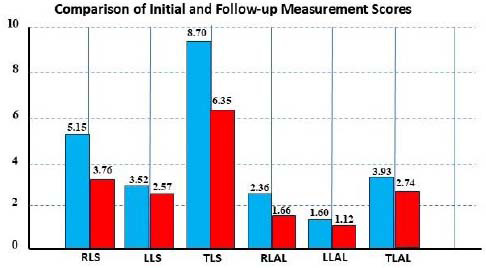
The X-axis represents the time points (Initial *vs*. Follow-up), and the Y-axis shows quantitative lung severity scores and the number of affected lobes. Blue bars indicate the initial measurements; red bars indicate follow-up measurements. **Note:** (RLS: Right Lung Score, LLS: Left Lung Score, TLS: Total Lung Score, RLAL: Right Lung Affected Lobes, LLAL: Left Lung Affected Lobes, TLAL: Total Lung Affected Lobes).

These results reflect the improvements in treatment outcomes and the patients’ recovery. A significant reduction in the right and left lung scores during follow-up measurements indicates a general improvement in the patients’ conditions (Fig. **2**).

### 3.5 Progression of Radiological Findings in COVID-19 Patients Over Time

During the initial evaluation, atelectasis was detected in 3% of the patients, increasing to 8% during the follow-up. Interstitial septal thickening, observed in 8% of the initial CT scans, rose to 25% in the follow-up scans. Fibrotic bands were present in 18% of the initial evaluations, increasing to 41% in the follow-up assessments. Pleural effusion was noted in 4% of the initial scans and 5% during follow-up.

These findings indicate a notable progression in the pathological changes observed on CT scans over time.

### 3.6 Univariate Analysis of Mortality

Binary logistic regression analysis identified the severity scale as the sole factor significantly influencing mortality. Increased severity scale scores correlated with higher mortality rates (Fig. **3**).

When analyzing the relationship between the severity scale and mortality, comparisons of initial and follow-up measurements revealed notable changes:

- The proportion of patients with Severity Scale 1 increased from 61% initially to 72% at follow-up.
- The proportion of Severity Scale 2 patients decreased from 27% to 18%.
- The proportion of Severity Scale 3 patients dropped from 12% to 10%.

Results indicate that higher severity scale scores significantly correlate with increased mortality rates. This highlights the critical impact of disease severity on patient outcomes.

### 3.7 Correlation Analysis Results

The correlation analysis examined relationships among variables, such as age, hospital stay duration, days between imaging, total score differences, and volume differences in the right and left lungs. Key findings are as follows (Table **4**):

- A positive correlation was found between age and total days of being hospitalized (r = 0.206, p = 0.020), indicating longer hospital stays with increasing age.
- A negative correlation was observed between age and total lung volume difference (r = -0.177, p = 0.047), suggesting smaller volume changes with advancing age.
- Similarly, a negative correlation was found between age and right lung volume difference (r = -0.218, p = 0.014), showing reduced improvement in right lung volume with age.

These findings emphasize the impact of age on clinical and volumetric parameters. Older patients experienced longer hospital stays and less significant lung volume recovery. This underscores the importance of careful monitoring and management in elderly COVID-19 patients.

## 4 DISCUSSION

This study evaluates the long-term changes in lung function and radiological findings during the recovery process from COVID-19, highlighting the persistent pulmonary effects of the disease. Our findings contribute to understanding the late-stage impacts of COVID-19 and emphasize the need for long-term follow-up.

Our results demonstrate that fibrosis-like opacities, consolidations, and interstitial thickening persisted even during the recovery phase in COVID-19 survivors. These changes were more pronounced in patients with severe symptoms requiring intensive care. Similar findings have been reported in long-term follow-up studies, which showed permanent parenchymal changes post-COVID-19 [21, 22].

The increased risk of pulmonary fibrosis post-COVID-19, widely observed in the literature, is associated with the progression of structural changes in the lungs [23]. Follow-up imaging revealed the persistence of fibrosis-like changes and interstitial thickening, suggesting that post-COVID-19 pulmonary fibrosis may be permanent and requires long-term monitoring [24]. These findings highlight the need for radiological and functional monitoring even after symptomatic recovery.

One of the most striking findings in this study was the significant increase in pulmo sinister volume compared to pulmo dexter. In our study, a significant increase in left lung volume was observed, whereas no significant change was detected in the right lung. This finding aligns with previous literature on the differential effects of COVID-19 on lung lobes. In particular, the anatomical and physiological characteristics of the left lung may contribute to its distinct response during the recovery process [25]. Several anatomical and physiological factors may contribute to this difference. Bronchus Principalis Sinister is longer and narrower than Bronchus Principalis Dexter, which may potentially affect the distribution of inhaled infectious particles. Additionally, pulmonary blood flow is not evenly distributed between the lungs, and the pulmo sinister receives slightly lower perfusion [26]. This may influence post-inflammatory healing patterns. Furthermore, the existing literature suggests that COVID-19 primarily affects the lower lobes, and since the pulmo sinister consists of only two lobes, these changes may appear more prominent in volumetric analyses [12, 27-29].

The data from this study reveal the significant role of age in the long-term effects of COVID-19 on lung function. Correlation analyses showed that as age increased, improvements in lung volume (both right and left) decreased, and hospital stays lengthened. These findings align with studies by Pan and Wu, which linked age-related lung function decline to diminished recovery rates [8, 30, 31].

Our volumetric results align with the findings of Bellini *et al.*, who observed limited recovery in lung volume in post-COVID-19 patients, particularly in younger individuals using multidetector CT imaging [12]. Similarly, Pan *et al.* and Otake *et al.* reported variable improvement in total lung volume over a 1 to 3-month period after infection [8, 29]. Our study demonstrated a statistically significant increase in left lung volume (p = 0.038), while right lung volume changes were not significant, which is consistent with findings suggesting asymmetric lung involvement during and after infection. In terms of radiological scoring, our results showed significant reductions in GGO/consolidation scores and affected lobes (Table **2**), consistent with the longitudinal improvements noted in Han *et al.* and Wu *et al*. [21, 31]. These findings reinforce the gradual but incomplete pulmonary recovery in a subset of patients.

Similar studies, such as those by Bellini *et al.*, emphasize limited lung volume recovery in post-COVID-19 patients, reinforcing the necessity for extended pulmonary follow-up [12]. These findings corroborate our results and provide critical evidence of permanent lung damage in elderly populations.

The significant association between disease severity and mortality in this study aligns with reports from patients experiencing severe symptoms requiring high inflammatory marker levels and intensive care. Disease severity increases the risk of persistent lung damage and fibrotic changes, underscoring the importance of advanced follow-up and antifibrotic treatment options for severely affected patients [9, 23, 32].

Recent studies have demonstrated the potential benefits of antifibrotic therapies, such as nintedanib and pirfenidone, in improving pulmonary function and slowing fibrotic progression in post-COVID-19 patients [33, 34]. The impact of antifibrotic therapies on post-COVID-19 fibrosis remains an area of ongoing investigation.

A meta-analysis reported that while antifibrotic drugs may improve lung function, they do not have a significant effect on chest CT scores, hospital stay duration, or mortality rates [35]. Similarly, a randomized trial comparing nintedanib and pirfenidone found that both treatments enhanced lung function, with nintedanib showing a greater impact on exercise capacity but with higher adverse effects [33, 36]. These findings indicate that high-risk groups, including elderly patients, smokers, and individuals with pre-existing lung conditions such as COPD or interstitial lung disease, may particularly benefit from antifibrotic interventions [33].

However, given the variability in treatment responses, antifibrotic therapies should be considered as part of a multidisciplinary approach rather than a universal post-COVID therapy. Recent findings suggest that while these treatments may slow fibrotic progression, they do not uniformly resolve radiological fibrosis, emphasizing the need for personalized treatment strategies. Further prospective clinical trials are needed to establish standardized treatment protocols and determine the most suitable candidates for these interventions [35].

The segmentation data obtained using the 3D Slicer software in this study is directly dependent on the quality of radiological images. To enhance the accuracy of the segmentation process and obtain more reliable volume measurements, the use of artificial intelligence-based image processing techniques is considered an important research area for the future. In recent years, deep learning-based noise reduction techniques have provided remarkable advancements in medical imaging analysis. The integration of such advanced technologies may enable a more precise analysis of long-term lung changes [37].

### 4.1 Limitations and Future Research

This study has several limitations. The retrospective design restricts causal inferences, and the absence of a control group limits comparison with unaffected individuals. The follow-up period of 54 days may not capture long-term changes, and factors such as smoking, obesity, and comorbidities were not consistently recorded. Fibrosis quantification was also not performed. Due to confidentiality policies, raw patient data cannot be shared.

Future research should involve larger, prospective studies with longer follow-up to explore the long-term pulmonary effects of COVID-19. Additionally, further investigation into antifibrotic therapies could help mitigate post-COVID complications, especially in high-risk groups.

## CONCLUSION

This study demonstrates the long-term effects of COVID-19 on lung function, highlighting persistent changes in both radiological and volumetric findings. The results emphasize the need for careful planning of follow-up and treatment strategies, particularly for elderly and severely symptomatic patients.

A multidisciplinary approach is crucial for understanding and addressing the long-term pulmonary impacts of COVID-19, enhancing patient recovery, and reducing the burden on healthcare systems. Evaluating post-COVID-19 follow-up strategies and antifibrotic treatment options is essential for improving patient quality of life and minimizing healthcare system challenges.

Pulmonary fibrosis is a significant long-term consequence of COVID-19. Our study demonstrated a significant increase in left lung volume and the persistence of fibrotic changes. However, current evidence on the effectiveness of antifibrotic therapies remains limited, highlighting the need for further large-scale studies. Therefore, long-term follow-up and the development of appropriate treatment strategies are essential for post-COVID-19 patients.

## AUTHORS’ CONTRIBUTIONS

S.A, O.M, S.H and T.M: Data analysis or interpretation; S.Y: Study conceptor design. It is hereby acknowledged that all authors have accepted responsibility for the manuscript's content and consented to its submission. They have meticulously reviewed all results and unanimously approved the final version of the manuscript.

## Figures and Tables

**Table 1:** Comparison of mean lung volumes calculated with 3D Slicer software during initial and follow-up measurements (n = 127).

**Variables**	**Initial Measurement (x̄±SD)**	**Follow-Up Measurement (x̄±SD)**	**t**	**p**
Right lung total volume (ml)	1930.38±685.06	1995.21±710.39	-1.558	0.122
Right lung COVID-19-affected volume (ml)	815.96±334.280	798.60±292.32	0.752	0.453
Left lung total volume (ml)	1703.24±597.20	1787.48±689.11	-2.095	**0.038**
Left lung COVID-19-affected volume (ml)	705.52±291.63	718.93±292.59	-0.543	0.588
Total COVID-19-affected lung volume (ml)	1521.47±605.61	1517.53±560.35	0.088	0.930
Total lung volume (ml)	3633.61±1250.00	3782.70±1353.85	-1.956	0.053

t: Paired-Samples T Test.

**Table 2:** Comparison of scoring (severity scale and number of affected lobes) during initial and follow-up measurements (n = 127).

**Variables**	**Initial Measurement (x̄±SD)**	**Follow-Up Measurement (x̄±SD)**	**t**	**p**
**Score**				
Right lung	5.15±3.56	3.76±4.19	4.595	**0.000**
Left lung	3.52±2.42	2.57±2.94	4.398	**0.000**
Total lung	8.70±5.88	6.35±7.07	4.665	**0.000**
**Number of Affected Lobes**				
Right lung	2.36±1.08	1.66±1.40	5.446	**0.000**
Left lung	1.60±0.71	1.12±0.94	5.973	**0.000**
Total	3.93±1.75	2.74±2.29	5.736	**0.000**

t: Paired-Samples T Test.

**Table 3:** Univariate analysis results of mortality.

**Variables**	**p**	**OR**	**95% CI for OR**
Age	0,127	1.029	0,992-1,067
Gender	0,865	1.106	0,348-3,518
Total lung volume difference	0,063	0,999	0,999-1,000
Total COVID-19-affected volume difference	0,374	1.000	0,999-1,002
Severity scale difference	0,032	2.725	1.093-6.797

**Table 4:** Correlation analysis results.

**Variables**	**Correlation Coefficient (r)**	**p-value**	**N**
Age and days in ward	0,169	0,058	127
Age and total days hospitalized	0,206*	0,020	127
Age and days between scans	0,033	0,713	127
Age and total score difference	-0,068	0,445	127
Age and right lung score difference	-0,044	0,621	127
Age and severity scale difference	-0,149	0,094	127
Age and total lung volume difference	-0,177*	0,047	127
Age and total COVID-19-affected volume difference	0,037	0,676	127
Age and left lung total volume difference	-0,110	0,218	127
Age and COVID-19-affected right lung volume difference	0,034	0,703	127
Age and right lung total volume difference	-0,218*	0,014	127

p < 0.05.

## Data Availability

The data and supportive information are available within the article.

## LIST OF ABBREVIATIONS

COVID-19: Coronavirus Disease 2019
PHEIC: Public Health Emergency of International Concern
COPD: chronic obstructive pulmonary disease

## STANDARDS OF REPORTING

STROBE guidelines were followed.

## References

[1] Coronavirus W.H.O. (2021). COVID-19 Cases, World.. https://covid19.who.int/.

[2] Li Q., Guan X., Wu P., Wang X., Zhou L., Tong Y., Ren R., Leung K.S.M., Lau E.H.Y., Wong J.Y., Xing X., Xiang N., Wu Y., Li C., Chen Q., Li D., Liu T., Zhao J., Liu M., Tu W., Chen C., Jin L., Yang R., Wang Q., Zhou S., Wang R., Liu H., Luo Y., Liu Y., Shao G., Li H., Tao Z., Yang Y., Deng Z., Liu B., Ma Z., Zhang Y., Shi G., Lam T.T.Y., Wu J.T., Gao G.F., Cowling B.J., Yang B., Leung G.M., Feng Z. (2020). Early Transmission Dynamics in Wuhan, China, of Novel Coronavirus–Infected Pneumonia.. N. Engl. J. Med..

[3] Jiang S., Xia S., Ying T., Lu L. (2020). A novel coronavirus (2019-nCoV) causing pneumonia-associated respiratory syndrome.. Cell. Mol. Immunol..

[4] Kwee T.C., Kwee R.M. (2020). Chest CT in COVID-19: What the Radiologist Needs to Know.. Radiographics.

[5] Li X., Zhao Y., Lu Y., Zheng Y., Mei N., Han Q., Ruan Z., Xiao A., Qiu X., Wang D., Yin B. (2022). Performances of clinical characteristics and radiological findings in identifying COVID-19 from suspected cases.. BMC Med. Imaging.

[6] Pontone G., Scafuri S., Mancini M.E., Agalbato C., Guglielmo M., Baggiano A., Muscogiuri G., Fusini L., Andreini D., Mushtaq S., Conte E., Annoni A., Formenti A., Gennari A.G., Guaricci A.I., Rabbat M.R., Pompilio G., Pepi M., Rossi A. (2021). Role of computed tomography in COVID-19.. J. Cardiovasc. Comput. Tomogr..

[7] Stevens R., John Mosley R. (2006). Gray’s Anatomy for Students.. Ann. R. Coll. Surg. Engl..

[8] Pan F., Ye T., Sun P., Gui S., Liang B., Li L., Zheng D., Wang J., Hesketh R.L., Yang L., Zheng C. (2020). Time course of lung changes at chest CT during recovery from Coronavirus disease 2019 (COVID-19).. Radiology.

[9] Özkan F., Öztürk M., Ödek Ö., Savaş M. (2023). Chronic Disease and Other Determinants in Deaths Due to COVID-19 From a Health Protection and Promotion Perspective: A Retrospective Analysis.. Disaster Med. Public Health Prep..

[10] Yong S.J. (2021). Long COVID or post-COVID-19 syndrome: putative pathophysiology, risk factors, and treatments.. Infect. Dis. Lond..

[11] Blanco J.R., Cobos-Ceballos M.J., Navarro F., Sanjoaquin I., Arnaiz de las Revillas F., Bernal E., Buzon-Martin L., Viribay M., Romero L., Espejo-Perez S., Valencia B., Ibañez D., Ferrer-Pargada D., Malia D., Gutierrez-Herrero F.G., Olalla J., Jurado-Gamez B., Ugedo J. (2021). Pulmonary long-term consequences of COVID-19 infections after hospital discharge.. Clin. Microbiol. Infect..

[12] Bellini D., Capodiferro P., Vicini S., Rengo M., Carbone I. (2023). Long COVID in young patients: Impact on lung volume evaluated using multidetector CT.. Tomography.

[13] Huang Y., Tan C., Wu J., Chen M., Wang Z., Luo L., Zhou X., Liu X., Huang X., Yuan S., Chen C., Gao F., Huang J., Shan H., Liu J. (2020). Impact of coronavirus disease 2019 on pulmonary function in early convalescence phase.. Respir. Res..

[14] Wang Y., Dong C., Hu Y., Li C., Ren Q., Zhang X., Shi H., Zhou M. (2020). Temporal Changes of CT Findings in 90 Patients with COVID-19 Pneumonia: A Longitudinal Study.. Radiology.

[15] Alrajhi N.N. (2023). Post-COVID-19 pulmonary fibrosis: An ongoing concern.. Ann. Thorac. Med..

[16] Hofmanninger J., Prayer F., Pan J., Röhrich S., Prosch H., Langs G. (2020). Automatic lung segmentation in routine imaging is primarily a data diversity problem, not a methodology problem.. Eur. Radiol. Exp..

[17] Fillion-Robin J.C., Pujol S., Bauer C., Jennings D., Fennessy F., Sonka M., Buatti J., Aylward S., Miller J.V., Pieper S., Kikinis R. (2012). 3D Slicer as an image computing platform for the quantitative imaging network.. Magn. Reson. Imaging.

[18] Hofmanninger J., Prayer F., Pan J., Röhrich S., Prosch H., G Langs. ( 2025). Automatic lung segmentation in routine imaging is primarily a data diversity problem, not a methodology problem.. Eur. Radiol. Exp..

[19] Chung M., Bernheim A., Mei X., Zhang N., Huang M., Zeng X., Cui J., Xu W., Yang Y., Fayad Z.A., Jacobi A., Li K., Li S., Shan H. (2020). CT Imaging Features of 2019 Novel Coronavirus (2019-nCoV).. Radiology.

[20] Malpani Dhoot N., Goenka U., Ghosh S., Jajodia S., Chand R., Majumdar S., Ramasubban S. (2020). Assigning computed tomography involvement score in COVID-19 patients: prognosis prediction and impact on management.. BJR Open.

[21] Han X., Chen L., Guo L., Wu L., Alwalid O., Liu J., Zheng Y., Chen L., Wu W., Li H., Luo Q., Zhao H., Zhang L., Bai Y., Sun B., Sun T., Gui Y., Nie T., Chen L., Yang F., Fan Y., Shi H., Zheng C. (2024). Long-term radiological and pulmonary function abnormalities at 3 years after COVID-19 hospitalisation: a longitudinal cohort study.. Eur. Respir. J..

[22] Zhang P, Li J, Liu H (2020). Long-term bone and lung consequences associated with hospital-acquired severe acute respiratory syndrome: a 15-year follow-up from a prospective cohort study.. Bone Research.

[23] Kim J., Chae G., Kim W.Y., Chung C.R., Cho Y.J., Lee J., Jegal Y., Joh J.S., Park T.Y., Hwang J.H., Nam B.D., Yoon H.Y., Song J.W. (2024). Pulmonary fibrosis followed by severe pneumonia in patients with COVID-19 infection requiring mechanical ventilation: a prospective multicentre study.. BMJ Open Respir. Res..

[24] Han X., Fan Y., Alwalid O., Zhang X., Jia X., Zheng Y., Shi H. (2021). Fibrotic Interstitial Lung Abnormalities at 1-year Follow-up CT after Severe COVID-19.. Radiology.

[25] Foti G, Longo C, Faccioli N, Guerriero M, Stefanini F, Buonfrate D. (2023). Quantitative assessment of lung volumes and enhancement in patients with COVID-19: Role of dual-energy CT.. Diagnostics.

[26] Cheng C.P., Taur A.S., Lee G.S., Goris M.L., Feinstein J.A. (2006). Relative lung perfusion distribution in normal lung scans: observations and clinical implications.. Congenit. Heart Dis..

[27] Li J., Yu X., Hu S., Lin Z., Xiong N., Gao Y. (2020). COVID-19 targets the right lung.. Crit. Care.

[28] Fahrni G., Rocha A.C., Gudmundsson L., Pozzessere C., Qanadli S.D., Rotzinger D.C. (2023). Impact of COVID-19 pneumonia on pulmonary vascular volume.. Front. Med. (Lausanne).

[29] Otake S., Shiraishi Y., Chubachi S., Tanabe N., Maetani T., Asakura T., Namkoong H., Shimada T., Azekawa S., Nakagawara K., Tanaka H., Fukushima T., Watase M., Terai H., Sasaki M., Ueda S., Kato Y., Harada N., Suzuki S., Yoshida S., Tateno H., Yamada Y., Jinzaki M., Hirai T., Okada Y., Koike R., Ishii M., Hasegawa N., Kimura A., Imoto S., Miyano S., Ogawa S., Kanai T., Fukunaga K. (2024). Lung volume measurement using chest CT in COVID-19 patients: a cohort study in Japan.. BMJ Open Respir. Res..

[30] Wu J., Liu J., Zhao X., Liu C., Wang W., Wang D., Xu W., Zhang C., Yu J., Jiang B., Cao H., Li L. (2020). Clinical Characteristics of Imported Cases of Coronavirus Disease 2019 (COVID-19) in Jiangsu Province: A Multicenter Descriptive Study.. Clin. Infect. Dis..

[31] Wu Q., Zhong L., Li H., Guo J., Li Y., Hou X., Yang F., Xie Y., Li L., Xing Z. (2021). A follow-up study of lung function and chest computed tomography at 6 months after discharge in patients with Coronavirus Disease 2019.. Can. Respir. J..

[32] Zhou M., Dong C., Li C., Wang Y., Liao H., Shi H., Lin A.P., Wang J., Hu Y., Zheng C. (2021). Longitudinal changes in COVID-19 clinical measures and correlation with the extent of CT lung abnormalities.. Int. J. Med. Sci..

[33] Al-kuraishy H.M., Batiha G.E.S., Faidah H., Al-Gareeb A.I., Saad H.M., Simal-Gandara J. (2022). Pirfenidone and post-Covid-19 pulmonary fibrosis: invoked again for realistic goals.. Inflammopharmacology.

[34] Saiphoklang N., Patanayindee P., Ruchiwit P. (2022). The Effect of Nintedanib in Post-COVID-19 Lung Fibrosis: An Observational Study.. Crit. Care Res. Pract..

[35] Shu Y., He L., Liu C. (2024). Impact of anti-fibrotic medications on post-COVID-19 pulmonary fibrosis: A systematic review and meta-analysis.. Int. J. Infect. Dis..

[36] Kerget B., Çil G., Araz Ö., Alper F., Akgün M. (2023). Comparison of two antifibrotic treatments for lung fibrosis in post-COVID-19 syndrome: A randomized, prospective study.. Med. Clín. Engl. Ed..

[37] Naqvi RA, Haider A, Kim HS, Jeong D, Lee SW (2024). Transformative Noise Reduction: Leveraging a transformer-based deep network for medical image denoising.. Mathematics.

