# Corrigendum to: The Long-term Volumetric and Radiological Changes of COVID-19 on Lung Anatomy: A Quantitative Assessment

**DOI:** 10.2174/157340562101251015120737

**Published:** 2025-10-15

**Authors:** A. Savranlar, M. Öztürk, H. Sipahioğlu, Y. Savranlar, M. Tahta Şahingöz

**Affiliations:** 1 Department of Radiology, Kayseri City Training and Research Hospital, Kayseri, Türkiye; 2 Department of Anatomy, Kayseri City Training and Research Hospital, Kayseri, Türkiye; 3 Department of Intensive Care, Kayseri City Training and Research Hospital, Kayseri, Türkiye; 4 Department of Medical Histology and Embryology, Kayseri City Training and Research Hospital, Kayseri, Türkiye; 5 Department of First and Emergency Aid Program, Niğde Ömer Halisdemir University, Niğde, Türkiye

In the online version of the article, a change was made in the affiliation of Dr. M. Tahta Şahingöz of the article entitled “The Long-term Volumetric and Radiological Changes of COVID-19 on Lung Anatomy: A Quantitative Assessment” has been updated in “Current Medical Imaging”, 2025; 21: e15734056372497 [1].

The original article can be found online at: https://www.eurekaselect.com/article/149638

**ORIGINAL:**

Tahta Şahingöz

Department of First and Emergency Aid Program, Niğde University, Niğde, Türkiye

**CORRECTED:**

Tahta Şahingöz

Department of First and Emergency Aid Program, Niğde Ömer Halisdemir University, Niğde, Türkiye

## References

[1] Savranlar A., Öztürk M., Sipahioğlu H., Savranlar Y., Tahta Şahingöz M. (2025). The Long-term Volumetric and Radiological Changes of COVID-19 on Lung Anatomy: A Quantitative Assessment.. Current Medical Imaging.

